# Incidence of adverse cardiovascular events in patients with insomnia: A systematic review and meta-analysis of real-world data

**DOI:** 10.1371/journal.pone.0291859

**Published:** 2023-09-21

**Authors:** Eman Ali, Asim Shaikh, Farah Yasmin, Fatima Sughra, Ayesha Sheikh, Rabia Owais, Hamna Raheel, Hafeez Ul Hassan Virk, Jihad A. Mustapha

**Affiliations:** 1 Dow Medical College, Dow University of Health Sciences, Karachi, Pakistan; 2 Aga Khan University Hospital, Karachi, Pakistan; 3 Yale University School of Medicine, New Haven, CT, United States of America; 4 Adena Regional Medical Center, Chillicothe, OH, United States of America; 5 Michigan State University College of Human Medicine, Grand Rapids, MI, United States of America; University of Catania, ITALY

## Abstract

Insomnia is a prevalent sleeping disorder associated with increasing cardiovascular (CV) mortality and morbidity. However, data incorporating recent clinical studies evaluating these outcomes is scarce. Hence, we aimed to investigate the association of insomnia with CV mortality, myocardial infarction (MI), all-cause mortality, and incidence of CV disease by conducting the first-ever meta-analysis of real-world data evaluating these CV outcomes. MEDLINE and Scopus databases were queried till August 2022 to identify studies comparing prespecified outcomes in patients with and without insomnia. The primary outcomes were CV mortality and myocardial infarction, while secondary outcomes included all-cause mortality, and CV-disease incidence. All data were pooled using an inverse-variance weighted random-effects model, and results were reported as relative risks (RRs) and p-values. 21 studies were analyzed. Risks for CV mortality and MI were significantly higher in patients with insomnia (RR 1.53, p<0.01, and RR 1.48, p = 0.03, respectively). The risk for all-cause mortality and CV disease incidence was also significantly higher in insomnia patients (RR 1.14, p = 0.03, and RR 1.31, p<0.01, respectively). Individuals with insomnia experience a higher risk of long-term mortality, MI, and incidence of CV disease.

## Introduction

Insomnia comprises one of the most frequent sleep-related disorders affecting up to 30% of the world’s population, and an alarming 44% of cardiovascular disease (CVD) patients [1–3]. It is characterized by difficulty in the initiation, duration, consolidation, or quality of sleep, despite adequate opportunity for sleep, resulting in some form of daytime impairment [4]. Some of the known risk factors for insomnia include hypertension, metabolic disorders such as diabetes mellitus (DM), and CVD [5]. Furthermore, delirium has been reported to have a bidirectional relationship with sleep/wake disruption, which positively correlates with aging. Parallel symptoms such as cognitive dysfunction, fluctuating periods of inattention, and mental status have been noted in sleep-deprived and delirious states [6]. The management of delirium in intensive care units (ICUs) is primarily focused on non-pharmacological modalities such as early mobilization, limiting the use of sedative drugs, and methods to improve sleep quality, as no drug has demonstrated clear benefits, despite the fact that several drugs have been investigated [7–9]. Even though chronic has been regarded as a separate disorder [9] comorbid insomnia and sleep apnea (COMISA) are the most prevalent co-occurring sleep disorders, with a global prevalence between 18% and 42% [10]. Robotic-assisted obstructive sleep apnea (OSA) surgeries show promise for improving insomnia associated with sleep apnea, more research is needed to determine if these procedures offer any clear advantages over traditional surgeries in alleviating insomnia symptoms and improving sleep quality and daytime functioning [11].

The evidence linking insomnia to hypertension (HTN) [12], coronary heart disease (CHD) [13], heart failure (HF) [14], cardiovascular (CV) mortality [15], and all-cause mortality [16] has been growing in the past decade. A recent retrospective analysis evaluating 330 patients with insomnia (9.7%), and 3084 patients without insomnia (90.3%) demonstrated a higher one-year all-cause mortality risk among patients with insomnia relative to those without (25.1% vs 16.2%, P = 0.001) [17]. Similar findings were reported by Mahmood et al. who followed 15,511 patients with insomnia for fourteen years, and showed an increased risk of all-cause mortality in this cohort. While these studies parallel the findings of the recent meta-analysis, some newly published studies have undermined the magnitude of these results after adjusting for confounders and covariates [18–21]. A very recently published study by Lechat et al. (2022) showed non-significant positive association between insomnia and coexisting obstructive sleep apnea with incidence of cardiovascular events (hazard ratio 1.38 with 95% CI [0.92–2.07]) after adjusting for pre-specified covariates [20]. Similarly, another recent study examining the risk of myocardial infarction (MI) in HIV patients with insomnia showed no significant association between type 1 MI and insomnia but a reduced yet significant association with type 2 MI after adjusting for confounders [21]. Observational studies examining comparative clinical outcomes in patients with and without insomnia have yielded conflicting and inconsistent results, and thus meriting the conduction of an updated meta-analysis to adequately address these queries which can have major impact on cardiovascular outcomes.

Few meta-analytic studies have been conducted which evaluated cardiovascular outcomes in patients with insomnia symptoms. However, these were inadequate as a meta-analysis published in 2014 evaluated cardiovascular outcomes in insomniac patients, but failed to categorize various components separately, and provided an estimated pooled effect size of associated conditions under a single outcome which did not comprehensively evaluate the impact of insomniac disorders on individual cardiovascular components such as MI, CV-mortality, CV-disease incidence, and all-cause mortality [22]. Another meta-analysis published in 2019 was also limited by a few clinical outcomes including risk of all-cause mortality, and CV-disease incidence with no real-world data [23]. Cohort studies with large-sample sizes, and longer follow-up periods have been published recently which further amplify the need for a newer meta-analysis that can evaluate the impact of insomnia on CVD.

## Methods

We conducted this systematic review and meta-analysis in concordance with guidelines provided by the Preferred Reporting Items for Systematic Reviews and Meta-Analysis statement (PRISMA) [24], and the Risk of Bias in Systematic reviews and Assessment of Multiple Systematic Reviews (AMSTAR) 2 [25]. This meta-analysis has been registered on Prospero (CRD42023442367).

### Literature search strategy

Two independent researchers (E.A and A.S) systematically searched major databases including MEDLINE/PubMed, Google Scholar, and Cochrane library from their inception till August 2022. The keywords used for conducting the search included “insomnia”, “sleep complaints”, “sleep initiation”, “sleep disorders”, “sleep disturbances”, “disorders of initiating and maintaining sleep”, “poor sleep quality”, “cardiovascular disease”, “myocardial infarction” and “mortality” along with incorporating relevant MesH terms and boolean operators. Reverse snowballing of previous meta-analyses was employed by searching through reference lists supplemented by no restrictions on time and language. PICOS criteria and different search strings designed for each database have been mentioned in **S1 and S2 Tables respectively in S1 File**. Other data sources were also searched namely bibliographies of editorials, conference proceedings of indexed abstracts, relevant reviews from major medical journals, and databases of grey/unpublished/unprinted literature.

### Study design and selection criteria

The method for eligibility and decision to include/exclude articles was hierarchical, based on a review of title, abstract and full-text. Joanna Briggs Institute’s (JBI) protocol was followed for study selection and critical appraisal which provides more specific and rigorous criteria for study selection process [26]. The pre-defined inclusion criteria were: 1) individuals over the age of 18; 2) observational studies (prospective and retrospective cohorts) and secondary analysis of original studies which reported only insomnia disorder or associated four major symptoms including difficulty in initiating sleep (DIS), difficulty in maintaining sleep (DIMS), non-restorative sleep (NRS), and early morning awakening (EMA); 3) provided one of the following outcomes pertaining to sleep complaints or insomnia symptoms: MI, CV-mortality, all-cause mortality, and CV-disease incidence. The pre-defined exclusion criteria included: 1) Studies which involved animals and minors; 2) Original studies including case reports, case series and case-control studies; and 3) Studies without comparison or control.

### Data extraction and assessment of quality

All articles retrieved from the systematic search were exported to EndNote X9 Reference Manager (Clarivate Analytics, Philadelphia, Pennsylvania) where duplicates were removed among different online databases. Two independent researchers (E.A and A.S) thoroughly evaluated the remaining articles, and only those studies that satisfied the pre-specified inclusion criteria were chosen. The titles and abstracts of all studies were initially screened, which was then followed by a full-text review of the article to determine its relevance. Any discrepancies were resolved by discussion with the third researcher (F.Y). We collected data for study characteristics including author, year of publication, region/hospital, study design, and follow-up period. Population characteristics including sample size, mean age, number and percentage of males and females, and baseline comorbidities including DM, and HTN were also abstracted for both insomnia and non-insomnia cohorts. The primary outcomes for this analysis were CV-mortality and MI. Other secondary outcomes included all-cause mortality, and CV-disease incidence. For those studies reporting distinct categories of insomnia, their events were separately merged under insomnia disorder. Two independent researchers (E.A and A.S) independently carried out the methodological quality assessment of the included observational studies using the Newcastle Ottawa Scale (NOS) which assesses the selection, comparability, and outcome assessment biases [27]. This scale has been well-established for its content validity and inter-rater reliability, relating to task of incorporating quality assessments in the interpretation of meta-analytic results [28]. A ’star system’ was used in which a study is judged on three broad perspectives: the selection of the study groups; the comparability of the groups; and the ascertainment of either the exposure or outcome of interest for case-control or cohort studies, respectively. Studies with a total score of 8 or 9 had a predicted low risk of bias, whereas studies with a total score of 6 or 7 had a predicted medium risk of bias. The investigators assessed the risk of bias for the included studies and assigned a score for each category. Further, the score assigned to each study was categorized into ratings as good, fair, or poor quality.

### Statistical analysis

All statistical analysis was carried out using Review Manager v.5.3 (version 5.4; Copenhagen: The Nordic Cochrane Centre, The Cochrane Collaboration, 2022). The results were presented as risks ratio (RRs) with 95% confidence intervals and pooled using an inverse variance weighted random effects model. For studies which reported separate adjusted effect sizes such as RR for different insomnia symptoms, we pooled RRs together to calculate average RR. The Higgins test (*I*^*2*^) was used to categorize heterogeneity as low (<25%), moderate (25–20%), and high (>50%) [29]. Sub-group analysis was also performed to determine the association of outcomes with follow-up period. For studies assessing all-cause mortality, insomnia patients were categorized into three sub-groups (up to 5 years, within 5–10 years and within 10–20 years). For studies assessing CV-disease incidence, insomnia patients were categorized into two sub-groups (up to 10 years and within 10–20 years). Following the elimination of study that contributed the most overall heterogeneity, sensitivity analysis was carried out for results with *I*^*2*^ > 50, and effect sizes were recalculated. To assess the impact of small-scale studies, publication bias was assessed via. a graphical representation of the funnel plot along with quantitative assessment using the Beggs, and Egger regression tests. A p-value ≤0.05 was considered significant in all cases.

## Results

**Fig 1:**
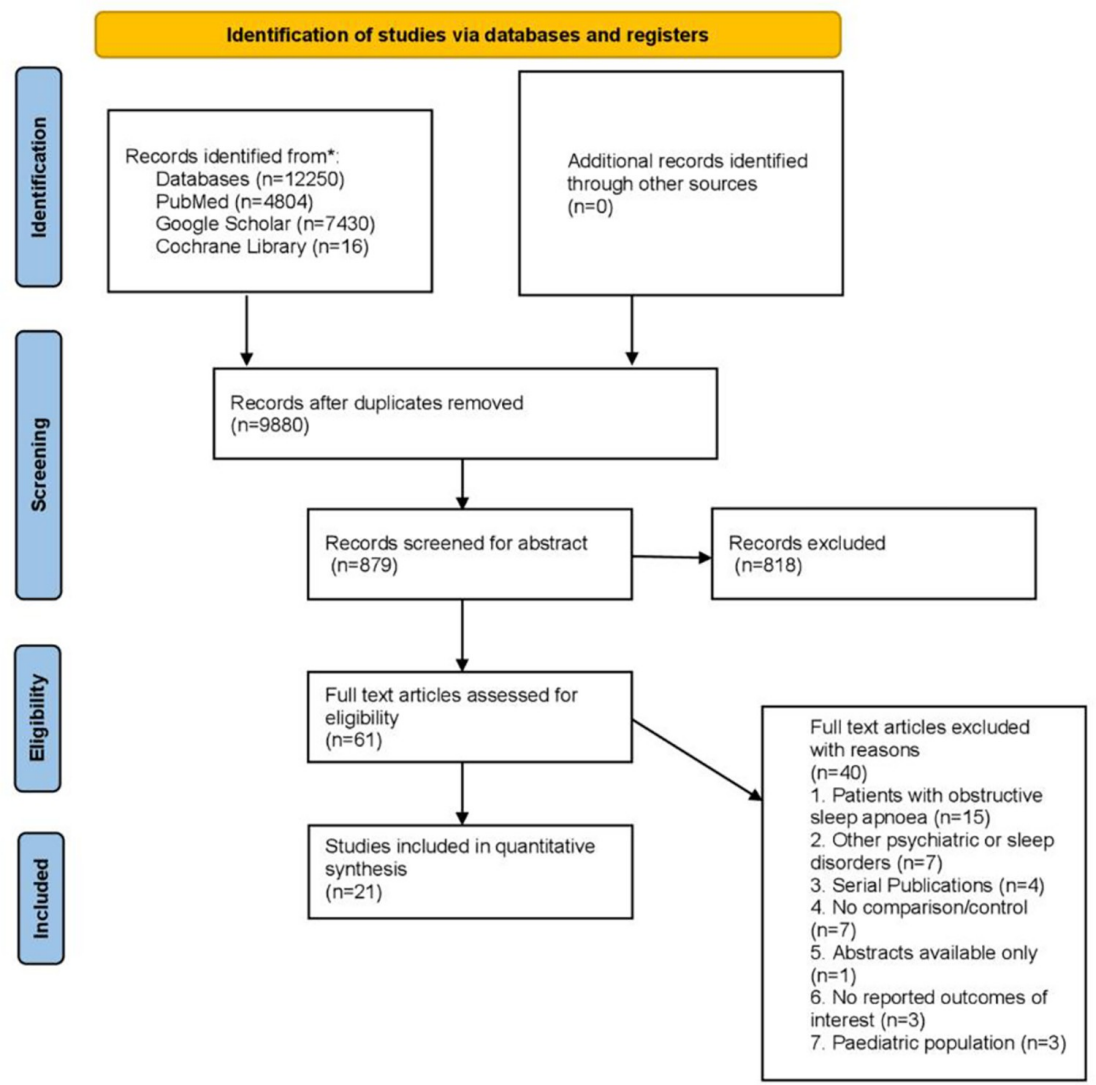
PRISMA flow diagram.

Our initial literature search of four databases yielded 12,250 results based on our search strategy out of which 9,980 citations remained after removing the duplicates. Screening based on title and abstract resulted in an exclusion of 881 articles. After screening full texts for eligibility, 40 articles were excluded. As a result, 21 observational studies [18, 20, 21, 30–47]. were selected for conducting our meta-analysis. The entire literature search process has been depicted by PRISMA flow diagram in **Fig 1**.

### Baseline characteristics and quality assessment

The detailed study and population characteristics of the included studies has been given in **S3 and S4 Tables in S1 File**. A total of 388,906 insomnia patients, and 2,194,211 healthy subjects were included in our analysis. A total of 4 studies reported MI, and 3 studies reported CV-mortality respectively. Other outcomes including all-cause mortality and CV-disease incidence were reported by 10 and 9 studies, respectively. The mean age of the study population was 59.4 years for the insomnia group, and 58.6 years for the control group. The follow-up duration in the included studies ranged from 3 years to 19.6 years. Detailed quality assessment of each study has been provided in **S5 Table in S1 File**. All included observational studies demonstrated a robust methodology, and were graded as ‘good quality’ based on the NOS.

### Results of meta-analysis

#### Primary outcomes

**Fig 2:**
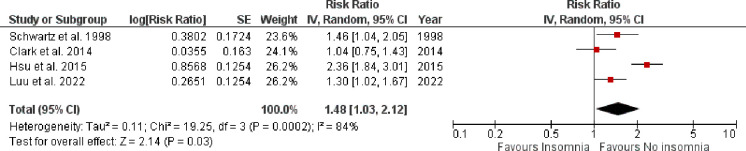
Forest plot evaluating the incidence of MI in patients with vs. without insomnia.

**Fig 3:**
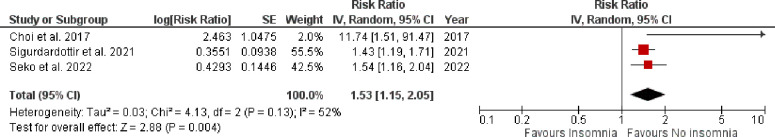
Forest plot evaluating CV-mortality in patients with vs. without insomnia.

Myocardial infarction was reported by four studies as shown in **Fig 2**. Pooled analysis revealed that the incidence of MI was significantly higher in patients with vs. without insomnia (RR: 1.48, 95% CI [1.03–2.12], p-value = 0.03, *I*^*2*^ = 84%). A total of 3 studies provided data on CV-mortality among insomnia patients as shown in **Fig 3**. Pooled analysis revealed that CV-mortality was significantly increased in insomnia group as compared to those without insomnia (RR: 1.53, 95% CI [1.15–2.05], p-value = 0.004, *I*^*2*^ = 52%).

#### Secondary outcomes

**Fig 4:**
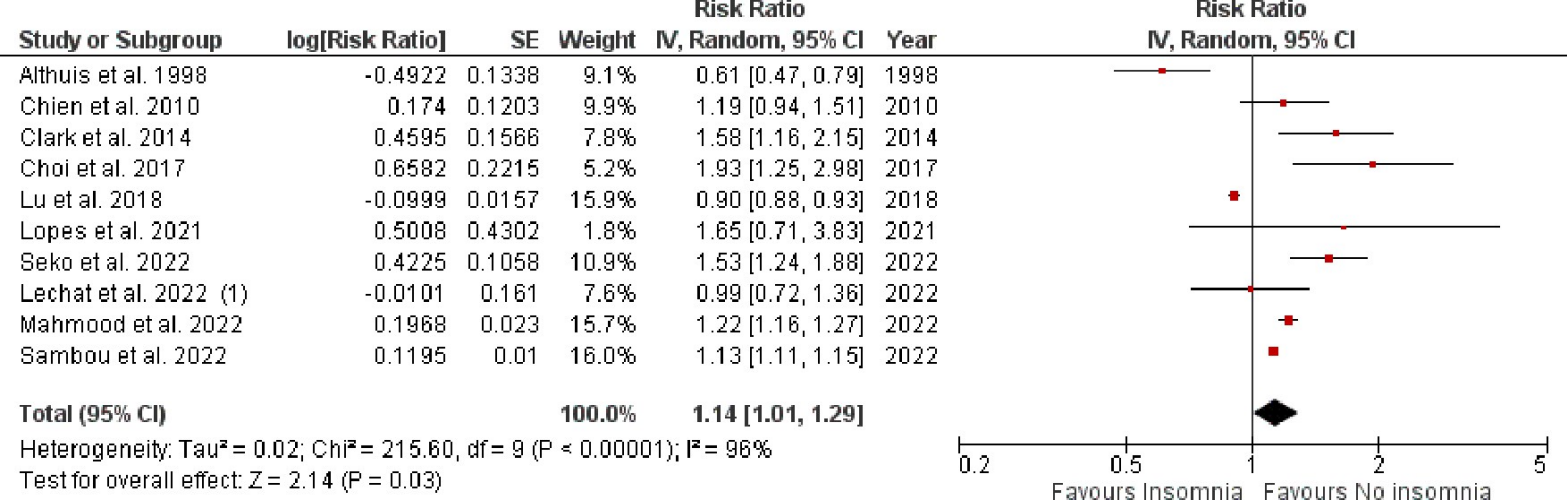
Forest plot evaluating all-cause mortality in patients with vs. without insomnia.

**Fig 5:**
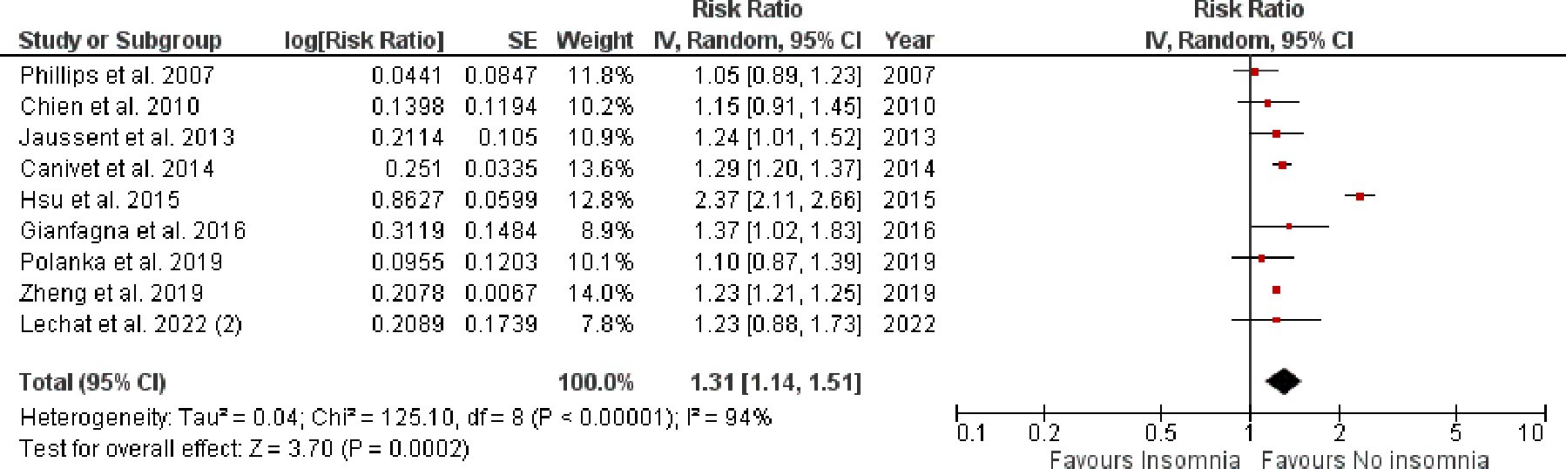
Forest plot evaluating the CV-disease incidence in patients with vs. without insomnia.

Pooled analysis of 10 observational studies reported a significantly higher rate of all-cause mortality in patients with vs. without insomnia (RR: 1.14, 95% CI [1.01–1.29], p-value = 0.03, *I*^*2*^ = 96%) as shown in **Fig 4**. A total of nine observational studies reported impact of insomnia on CV-disease incidence as shown in **Fig 5**. The pooled analysis revealed that the incidence of CV events was significantly higher in insomnia patients when compared with those without insomnia (RR: 1.31, 95% CI [1.14–1.51], p-value = 0.0002, *I*^2^ = 94%).

### Subgroup analysis

**Fig 6:**
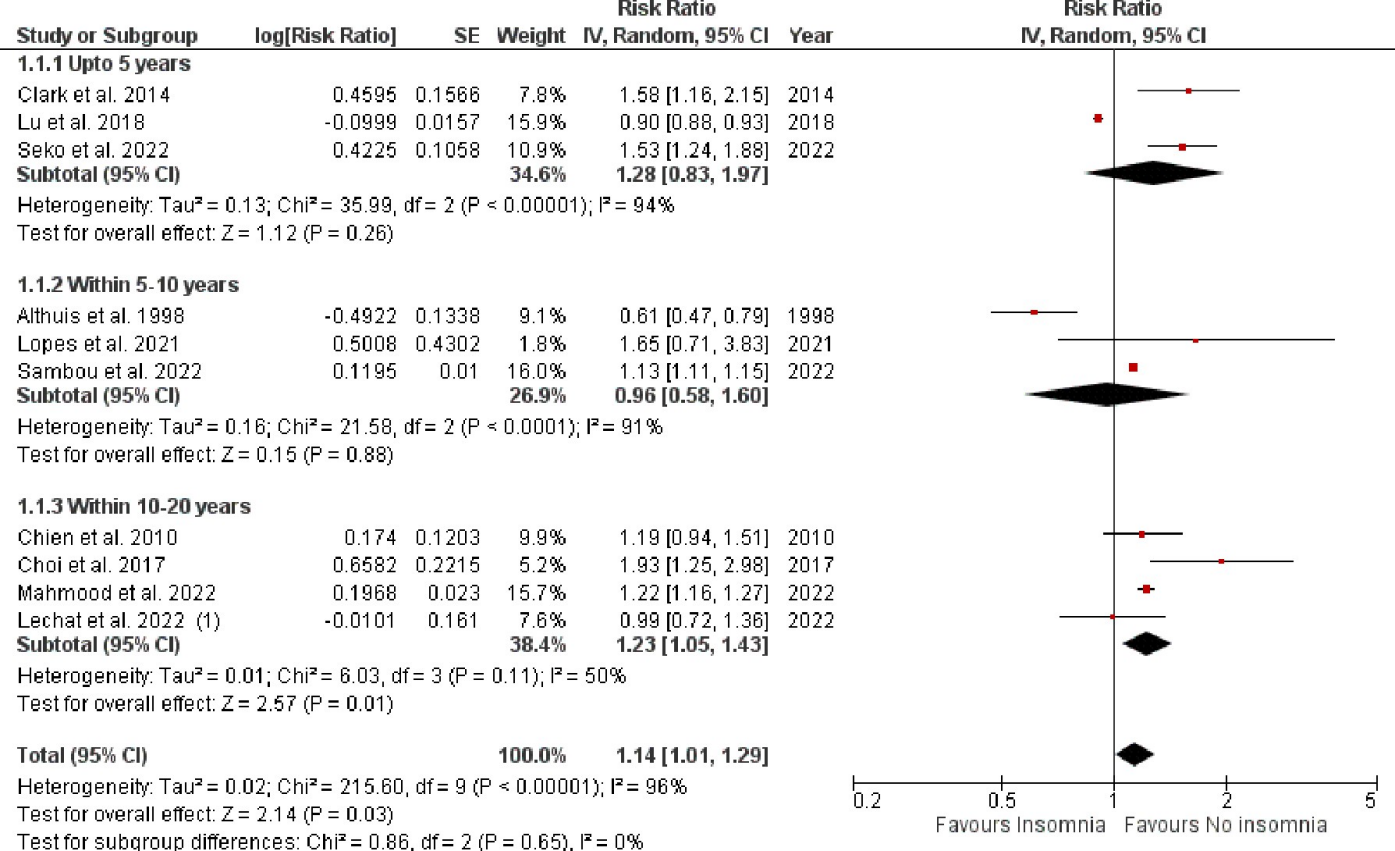
Sub-group analysis comparing all-cause mortality in patients with vs. without insomnia based on length of follow-up period.

**Fig 7:**
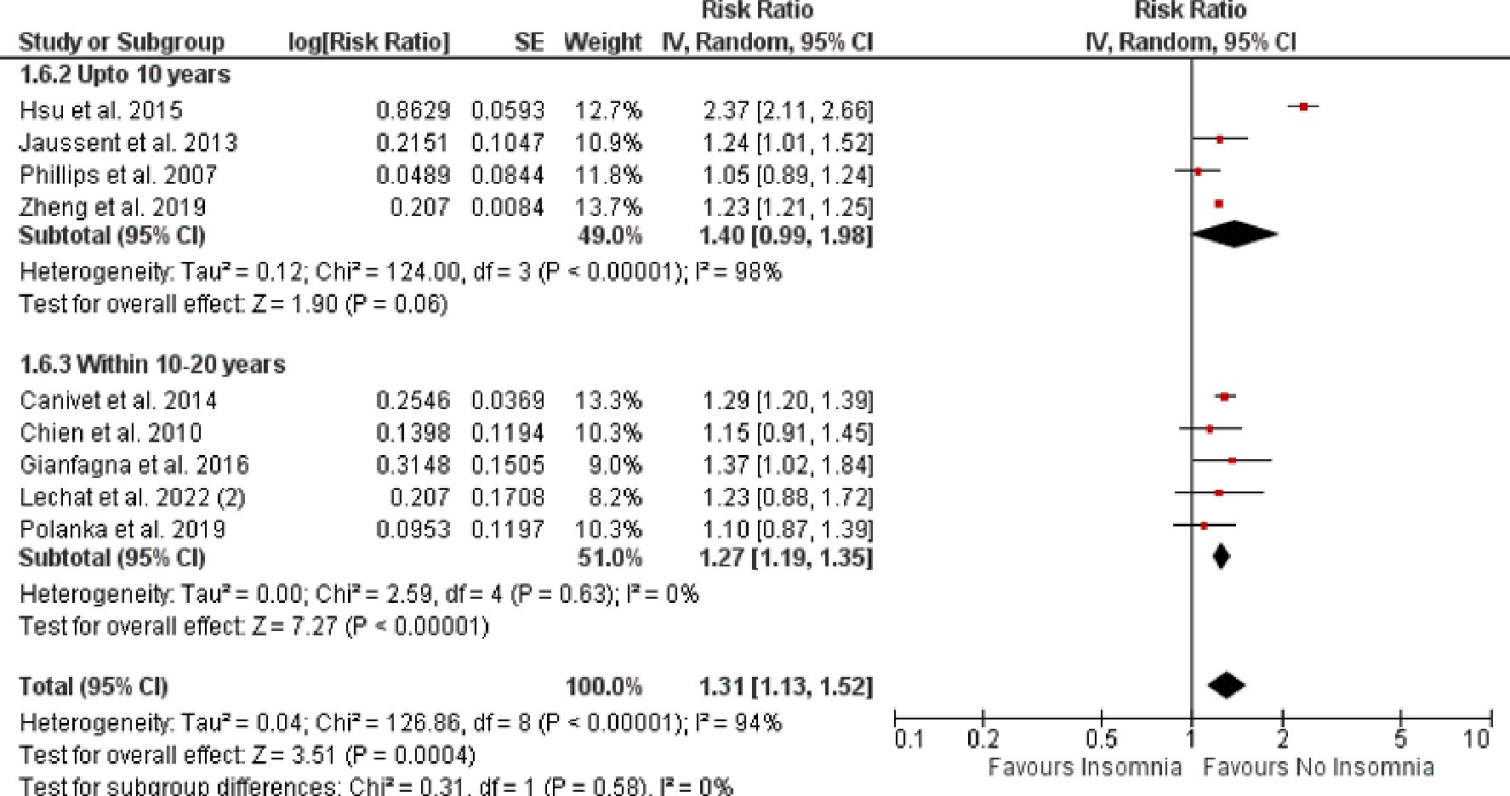
Sub-group analysis comparing CV-disease incidence in patients with vs. without insomnia based on length of follow-up period.

All-cause mortality was stratified based on the length of follow-up period, and demonstrated a statistically non-significant association with insomnia at <5 years (RR: 1.28, 95% CI [0.83–1.97], p-value = 0.26, *I*^*2*^ *=* 94%), and 5–10 years (RR: 0.96, 95% CI [0.58–1.60], p-value = 0.88, *I*^*2*^ *=* 91%) respectively. However, when analyzed for 10–20 years subgroup, all-cause mortality was noted to be significantly higher in the insomnia group vs. without insomnia (RR: 1.23, 95% CI [1.05–1.43], p-value = 0.01, *I*^*2*^ *=* 50%) as shown in **Fig 6**. For the outcome of CV-disease incidence, we evaluated that CV events increased non-significantly up to 10-years (RR: 1.40, 95% CI [0.99–1.98], p-value = 0.06, *I*^*2*^ *=* 98%). However, consistent trends with the overall result were observed at 10–20 years which revealed significantly higher incidence of CV events among insomnia patients vs. those without insomnia (RR: 1.27 95% CI [1.19–1.35], p-value<0.00001, *I*^*2*^ *=* 0%) as shown in **Fig 7**.

### Sensitivity analysis

A sensitivity analysis was conducted for all the primary and secondary outcomes including MI, all-cause mortality, and CV-disease incidence after identifying and excluding either retrospective or prospective studies with low-quality assessment scores or studies with different demographics and sample sizes. In the analysis of outcomes including MI and CV-disease incidence, excluding Hsu et al. (2015) which was underpowered for potential confounders (physical activity, smoking, body mass index, alcohol and caffeine consumption, dietary factors, and family history), caused the heterogeneity to decrease from 84% to 12% and 94% to 1% respectively **(S1 and S2 Figs in S1 File)**. In the outcome of all-cause mortality, excluding three studies Sambou et al. (2022) with selective study population i.e., Caucasian race and source of recall bias owing to self-reporting of insomnia symptoms, Lu et al. (2018) including patients with baseline chronic kidney disease (CKD), and Althuis et al. (1998) with low quality assessment score of 6 points, caused the heterogeneity to decrease from 96% to 55% (**S3 Fig in S1 File**).

### Publication bias

We evaluated publication bias for all outcomes studied in our meta-analysis as shown in **S6 Table in S1 File**. Beggs and Mazumdar’s test for rank correlation provided a p-value of 1.000, 0.2963, 0.4743, and 0.7545 for MI, CV-mortality, all-cause mortality, and CV-disease incidence respectively, indicating no evidence of publication bias. Similarly, Egger’s test for a regression intercept provided a p-value of 0.4904, 0.0662, 0.7585, and 0.2785 for MI, CV-mortality, all-cause mortality, and CV-disease incidence respectively, indicating no evidence of publication bias. The funnel plots showed no evidence of asymmetry as shown in **S4-S7 Figs in S1 File**.

## Discussion

Our analysis aimed to evaluate the association between insomnia and adverse cardiovascular outcomes, and reported that individuals with insomnia and insomnia-related symptoms have significantly increased risk of MI (48%), CV-mortality (53%), CV-disease incidence (14%), and all-cause mortality (31%) as compared to the healthy subjects. Insomnia, characterized as habitual quality of having difficulties in DIMS and often accompanied by daytime dysfunction (DDF), is the most common sleep disorder and the second most prevalent psychiatric disorder worldwide [48]. Emerging evidence from the vast medical literature including a majority of cohort and meta-analytic studies suggest a significant association between increased risk of CVDs, and insomnia-related symptoms. In 2014, Sofi et al. conducted a meta-analysis on the role of insomnia in development of cardiovascular mortality and cumulative analysis demonstrated a 45% increased risk of CVD incidence/cardiovascular mortality in insomniac patients when compared to healthy subjects [22]. In comparison to this meta-analysis which studied both outcomes cumulatively, we studied the pooled analysis of CV-disease incidence and CV-mortality individually and covered a broader range of cardiovascular related outcomes. According to research published in the European Journal of Preventive Cardiology, insomnia is considered as an established risk factor for MI [49]. Similarly, another meta-analysis conducted by Hu et al. paralleled our findings of 13% increased risk in the cumulative incidence of cardiocerebrovascular disease in insomniac patients [50].

Multiple studies have suggested insomnia as a risk factor for increased incidence of cardiovascular-related death and elevated risk of MI. Upon our pooled analysis, a 48% and 53% increased risk of MI and cardiovascular mortality in insomnia patients as compared to healthy subjects was observed respectively. Comparable to our results, a recently conducted meta-analysis by Dean et al. revealed significant association between insomnia and MI when compared with non-insomniacs (RR =  1.69, 95% [CI]  =  1.41–2.02, p < .00001) [51].

Pooled analysis of 9 studies reported 14% increased risk of all-cause mortality in our study. Our results were confirmed by a meta-analytic study by Ge at al. (2019) which showed that DFA and NRS were associated with an increased risk of all-cause mortality (DFA: HR: 1.13, 95% CI [1.03 to 1.23], p = 0.009, moderate certainty; NRS: HR: 1.23, 95% CI [1.07 to 1.42], p = 0.003, high certainty) [23]. Additionally, an interesting finding was observed in our sub-group analysis. Upon stratification based on mean follow-up duration of <5 or 5–10 years, all-cause mortality was not statistically significant. However, insomnia patients with mean follow-up duration of 10–20 years demonstrated findings that were consistent with the overall result indicating that significant increase in mortality rates were associated with longer follow-up periods. Increased risk of all-cause mortality in insomniac patients has been attributed to various theoretical models either from a physiological or psychological standpoint. Insomnia is a known independent risk factor for the onset of several psychological problems, including depression and anxiety [52–54]. The processes by which insomnia precedes the emergence of psychological disorders are unknown [55], however, it is hypothesized that the comorbidity of these diseases raises the risk of mortality in those who have insomnia. This is most likely caused by the higher-than-average rates of self-harm and suicide associated with certain psychological illnesses [56], however, additional research is needed.

Our meta-analysis also demonstrated a 31% increased CV-disease incidence among insomniac patients. This finding has been previously noted in a clinical trial conducted by Kanno et al. which evaluated the prognostic significance of insomnia among HF patients, and showed that the incidence of serious cardiac events was significantly higher among HF patients with insomnia. One potential explanation for this finding could be attributed to increased levels of renin, and aldosterone in this cohort due to an activated renin-angiotensinogen-aldosterone system (RAAS) thereby supporting the link between insomnia and serious cardiac events. Thus, it was demonstrated that HF patients with insomnia have activated RAAS and lower exercise capacity [57].

It has been well-established that the loss of synchronization between the body’s normal circadian rhythm and its functions as a result of shift work, which exposes a person to light at odd hours, and reduced or altered sleep patterns observed in sleep disorders like insomnia, and obstructive sleep apnea is associated with an elevated risk of CVDs [58, 59]. The subtype of insomnia (DFA or EMA) can impact the rate of co-occurrence with OSA [60]. Chung found that sleep maintenance and early morning awakenings were the most common subtype of insomnia associated with OSA [61]. Surgical procedures that enlarge the airway space through palatal stiffening operations and radiofrequency ablation of palate tissues have been shown to effectively relieve obstructive sleep apnea. The reduction in apnea-hypopnea events and arousals associated with these procedures often leads to improvements in sleep structure and continuity, thereby mitigating insomnia symptoms in many obstructive sleep apnea patients. Post-operative reductions in insomnia following palatal surgeries have been demonstrated using validated sleep and insomnia scales, indicating that these interventions can meaningfully improve sleep quality and daytime functioning in addition to resolving sleep-disordered breathing [62].

Our analysis further revealed that CV-disease incidence was significantly higher at longer follow-up durations i.e., >10 years as compared to shorter follow-ups. Chronic insomnia i.e., lasting for a duration of >1 year has been previously linked to HTN which was noted to be two times higher after a number of possible confounders were taken into consideration in a study (OR: 2.24; 95% CI [1.19–4.19]; p = 0.010) [63]. Many studies suggest HTN as a major risk factor for development of CVD regardless of the exact contribution. Hence, it may be noted that prevention of insomnia can reduce vascular consequences attributed to HTN. The majority of long-term consequences, including HF with and without preserved ejection fraction, atrial fibrillation, valvular heart disease, peripheral arterial disease, aortic syndromes, chronic kidney disease, end-stage renal disease, dementia, and Alzheimer’s disease are thought to be caused by HTN, according to consistent observational evidence [64]. It has been hypothesized that several mechanisms, including dysregulation of the hypothalamic-pituitary (HPA) axis [65], abnormal modulation of the autonomic nervous system, increased sympathetic nervous system (SNS) activity [66] increased systemic inflammation [63, 67], and increased atherogenesis [68, 69] underlie the association between insomnia and cardiovascular events.

Recent observational studies reported increased cardiovascular impairment, assessed by echocardiography, in patients affected by Coronavirus disease (COVID-19) [70–72]. As we have incorporated data published during the COVID-19 pandemic, we performed sensitivity analyses by excluding studies published amidst the pandemic, to rule out possible confounders in our study. We found a significant reduction in heterogeneity for all-cause mortality after excluding Sambou et al. 2022 suggesting COVID-19 might play role in abnormal cardiac function, however, additional research is needed. Exclusion of Hsu et al., Lu et al. and Althuis et al., led to a significant reduction in heterogeneity for MI, CV disease incidence, and all-cause mortality. We found that studies were subjected to various confounders including smoking, body mass index, alcohol and caffeine consumption, baseline comorbidities, selection, and recall biases. Current evidence suggests that CV disease can be caused by multiple risk factors including smoking tobacco, physical inactivity, poor eating habits, elevated blood pressure, type 2 diabetes, dyslipidemia, and obesity [73]. Thus, it can be inferred that these residual confounders could have been source of the heterogeneity in our study.

### Strengths and limitations

Our methodological approach comprised utilizing real-world data for our analysis with larger sample-sizes, and longer follow-up periods thereby increasing the statistical power of our findings, and enabling us to evaluate long-term impact of insomnia on adverse cardiovascular outcomes. Our study, however like any other study is not without certain limitations. First, the methods utilized to investigate insomnia in the included studies were rather diverse. This could have resulted in a non-homogeneous definition of insomnia across the studies. Second, the included studies showed significant clinical, and methodological heterogeneity. However, in order to overcome this, we conducted a sensitivity analysis for outcomes demonstrating high levels of heterogeneity excluding studies with high risk of bias, sample sizes, and varying demographical data. Third, the methods employed in our pooled studies to classify the presence/absence, intensity of insomnia and duration of follow-up also differed. To overcome this limitation, we restricted our inclusion criteria by including only four major insomnia symptoms, performed numerous sensitivity, and subgroup analyses, and used random-effects model to estimate the effect sizes. Additionally, the heterogeneity after leave-one-out analysis declined to near negligible states without impacting the significance and direction of the overall results, lending confidence to our findings. Lastly, the GRADE approach to rating certainty in evidence could support more cautious conclusions that consider key limitations in the body of evidence [74].

## Conclusion

There is an increased risk for serious cardiovascular events, and cardiovascular mortality in individuals with diagnosed insomnia disorder or those demonstrating symptoms of insomnia. In patients that are presented with such symptoms, clinicians should evaluate for cardiovascular risks and tailor interventions accordingly. Although focus on treating sleep disorders should be maintained, evaluating for additional coexisting cardiovascular risks such as coronary artery disease and smoking should influence therapy and clinical decision making. Adequately powered observational studies which are controlled for confounding risk factors and sleep disorders such as sleep apnea, and baseline comorbidities are required to provide a better understanding into pathophysiological mechanisms considering the substantial limitations and uncertainties in the current existing literature.

## Supporting information

S1 File (DOCX)
