# Supplementary material for: Incidence of adverse cardiovascular events in patients with insomnia: A systematic review and meta-analysis of real-world data
Source: PLoS One. 2023 Sep 21;18(9):e0291859. doi: 10.1371/journal.pone.0291859 (PMC10513332; doi:10.1371/journal.pone.0291859)
Supplement: S1 File — (DOCX) [file pone.0291859.s001.docx]

**Electronic Supplementary Material**

**Supporting information:**

**Supplementary Table 1:** PICOS criteria.

**Supplementary Table 2**: Search strategy used in each database searched.

**Supplementary Table 3:** Study characteristics of included studies.

**Supplementary Table 4:** Population Characteristics of included studies.

**Supplementary Table 5**: Quality assessment of observational studies using Newcastle Ottawa Scale.

**Supplementary Table 6:** Publication bias for outcomes.

**Supplementary Table 7:** Preferred Reporting Items for Systematic Reviews and Meta-analyses (PRISMA) checklist

**Supplementary Table 8:** AMSTAR-2 (Assessing the methodological quality of systematic reviews-2) Guidelines checklist.

**Supplementary Figure 1:** Forest plot of MI after excluding Hsu et al. (2015)

**Supplementary Figure 2:** All-cause mortality after excluding Sambou et al. (2022), Lu et al. (2018), and Althuis et al. (1998)

**Supplementary Figure 3:** CV-disease incidence after excluding Hsu et al. (2015)

**Supplementary Figure 4:** Funnel plot for MI in insomnia vs without insomnia.

**Supplementary Figure 5:** Funnel plot for CV-mortality in insomnia vs. without insomnia.

**Supplementary Figure 6**: Funnel pot for all-cause mortality in insomnia vs without insomnia.

**Supplementary Figure 7**: Funnel plot for CV-disease incidence in insomnia vs. without insomnia.

**Supplementary Table 1:** PICOS criteria.

| **P** | Adult population ≥ 18 |
| --- | --- |
| **I** | Insomnia and insomnia-related symptoms including difficulty in initiating sleep (DIS), difficulty in maintaining sleep (DIMS), non-restorative sleep (NRS), and early morning awakening (EMA) |
| **C** | Non-insomnia |
| **O** | MI, CV-mortality, all-cause mortality, CV-disease incidence |

**Supplementary Table 2:** Search strategy used in each database searched.

| Database | Search Strategy | Articles retrieved |
| --- | --- | --- |
| PubMed | ("insomnia s"[All Fields] OR "sleep initiation and maintenance disorders"[MeSH Terms] OR ("sleep"[All Fields] AND "initiation"[All Fields] AND "maintenance"[All Fields] AND "disorders"[All Fields]) OR "sleep initiation and maintenance disorders"[All Fields] OR "insomnia"[All Fields] OR "insomnias"[All Fields] OR (("sleep"[MeSH Terms] OR "sleep"[All Fields] OR "sleeping"[All Fields] OR "sleeps"[All Fields] OR "sleep s"[All Fields]) AND ("initial"[All Fields] OR "initially"[All Fields] OR "initials"[All Fields] OR "initiate"[All Fields] OR "initiated"[All Fields] OR "initiates"[All Fields] OR "initiating"[All Fields] OR "initiation"[All Fields] OR "initiations"[All Fields] OR "initiator"[All Fields] OR "initiators"[All Fields])) OR ("sleep initiation and maintenance disorders"[MeSH Terms] OR ("sleep"[All Fields] AND "initiation"[All Fields] AND "maintenance"[All Fields] AND "disorders"[All Fields]) OR "sleep initiation and maintenance disorders"[All Fields] OR ("disorders"[All Fields] AND "initiating"[All Fields] AND "maintaining"[All Fields] AND "sleep"[All Fields]) OR "disorders of initiating and maintaining sleep"[All Fields])) AND ("cardiovascular diseases"[MeSH Terms] OR ("cardiovascular"[All Fields] AND "diseases"[All Fields]) OR "cardiovascular diseases"[All Fields] OR ("cardiovascular"[All Fields] AND "disease"[All Fields]) OR "cardiovascular disease"[All Fields] OR ("myocardial infarction"[MeSH Terms] OR ("myocar("insomnia s"[All Fields] OR "sleep initiation and maintenance disorders"[MeSH Terms] OR ("sleep"[All Fields] AND "initiation"[All Fields] AND "maintenance"[All Fields] AND "disorders"[All Fields]) OR "sleep initiation and maintenance disorders"[All Fields] OR "insomnia"[All Fields] OR "insomnias"[All Fields] OR (("sleep"[MeSH Terms] OR "sleep"[All Fields] OR "sleeping"[All Fields] OR "sleeps"[All Fields] OR "sleep s"[All Fields]) AND ("initial"[All Fields] OR "initially"[All Fields] OR "initials"[All Fields] OR "initiate"[All Fields] OR "initiated"[All Fields] OR "initiates"[All Fields] OR "initiating"[All Fields] OR "initiation"[All Fields] OR "initiations"[All Fields] OR "initiator"[All Fields] OR "initiators"[All Fields])) OR ("sleep initiation and maintenance disorders"[MeSH Terms] OR ("sleep"[All Fields] AND "initiation"[All Fields] AND "maintenance"[All Fields] AND "disorders"[All Fields]) OR "sleep initiation and maintenance disorders"[All Fields] OR ("disorders"[All Fields] AND "initiating"[All Fields] AND "maintaining"[All Fields] AND "sleep"[All Fields]) OR "disorders of initiating and maintaining sleep"[All Fields])) AND ("cardiovascular diseases"[MeSH Terms] OR ("cardiovascular"[All Fields] AND "diseases"[All Fields]) OR "cardiovascular diseases"[All Fields] OR ("cardiovascular"[All Fields] AND "disease"[All Fields]) OR "cardiovascular disease"[All Fields] OR ("myocardial infarction"[MeSH Terms] OR ("myocardial"[All Fields] AND "infarction"[All Fields]) OR "myocardial infarction"[All Fields]) OR ("mortality"[MeSH Terms] OR "mortality"[All Fields] OR "mortalities"[All Fields] OR "mortality"[MeSH Subheading])) | 4804 |
| Cochrane Library | (“Insomnia” OR “sleep initiation” OR “disorders of initiating and maintaining sleep”) AND (“cardiovascular disease” OR “myocardial infarction” OR “mortality:) | 16 |
| Google Scholar | "Insomnia" AND "cardiovascular disease" AND "observational" AND "mortality" AND "myocardial infarction" | 7430 |

**Supplementary Table 3:** Study characteristics of included studies.

| **Author, year** | **Study type** | **Region/Hospital** | **Follow-up duration** |
| --- | --- | --- | --- |
| Althuis, 1998 [30] | Longitudinal study | Urban community consisting of 20 contiguous census tracts in Baltimore, Maryland | 6 years |
| Schwartz, 1998 [31] | Longitudinal study | North-central North Carolina. | 3 years |
| Philipps, 2007 [32] | Prospective, population-based study | 4 US communities: Forsyth County, NC (Winston- Salem); Jackson, MS; Washington County, MD. (Hagerstown); and suburbs of Minneapolis, MN. | 6 years |
| Chien, 2010 [33] | Prospective cohort study | Chin-Shan township 30 km north of metropolitan Taipei, Taiwan. | 15.9 years |
| Jaussent, 2013 [34] | Prospective study | Three-City Study, a multi-site longitudinal study involving three French cities, Bordeaux, Dijon and Montpellier. | 6 years |
| Canivet, 2014 [35] | Longitudinal register-based study | Malmö, Sweden. | 11 years |
| Clark, 2014 [36] | Prospective cohort study | Swedish Hospital, Sweden | 10 years |
| Hsu, 2015 [37] | Cohort study | Taiwan National Health Insurance Research Database | 10 years |
| Gianfagna, 2016 [38] | Cohort study | Monza, Brianza | 17 years |
| Choi, 2017 [39] | Cohort study | Center for Sleep and Chronobiology, Seoul National University Hospital, | 14 years |
| Lu, 2018 [40] | Cohort study | Department of Veterans Affairs, US | 4.76 |
| Polanka, 2019 [41] | Cohort study | VACS-survey cohort data | 10.8 years |
| Zheng, 2019 [42] | Cohort study | China Kadoorie Biobank, China | 9.6 years |
| Lopes, 2021 [43] | Prospective cohort | Campina Grande, state of Paraíba, Brazil | 5.3 years |
| Sigurdardottir 2021 [44] | Population based study | Nord-Trøndelag, Norway | 19.6 years |
| Lechat (1) 2022 [45] | Longitudinal study | Sleep Heart Health Study-Abbotsford, Victoria, Australia | 11 years |
| Mahmood, 2022 [18] | Prospective cohort | Health and Retirement Study in the United States. University of Michigan | 14 years |
| Lechat (2), 2022 [20] | Prospective | SHHS dataset from National Sleep Research Resource | 11.8 years |
| Seko, 2022 [46] | Prospective | Kyoto Congestive Heart Failure registry | 475 (IQR: 358–652) days |
| Sambou, 2022 [47] | Prospective | UK Biobank (UKB) | 6 years |
| Luu, 2022 [21] | Longitudinal cohort study | PWH at 5 Centers for AIDS Research Network of Integrated Clinical Systems sites | 4.4years |

**Supplementary Table 4:** Population characteristics of included studies.

|  | **Sample size** | | **Mean Age** | | **Population No. (%)** | | **DM No. (%)** | | **HTN. No (%)** | |
| --- | --- | --- | --- | --- | --- | --- | --- | --- | --- | --- |
| **Author (Year)** | **Insomnia** | **Non-insomnia** | **Insomnia** | **Non-insomnia** | **Female No. (%)** | **Male No. (%)** | **Insomnia** | **Non-insomnia** | **Insomnia** | **Non-insomnia** |
| Althuis, (1998) [30] | 442 | 336 | 74.1 | 73.7 | 778 (100) | 0 (0) | - | - | - | - |
| Schwartz (1998) [31] | 665 | 2291 | 73 | 73 | 1967 (66.5) | 993 (33.5) | 465 (15.7) | - | - | - |
| Philipps (2007) [32] | 4941 | 6922 | 54 | 54 | 4,815 (55.0) | 3,942 (45.0) | 836 (9.6) | 7,921 (90.4) | - | - |
| Chien (2010) [33] | 1318 | 2025 | 58.2 | 53.5 | 1754 (52.5) | 1589 (47.5) | 490 (14.3) | 429 (12.5) | 1320 (38.5) | 933 (27.2) |
| Jaussent (2013) [34] | 3491 | 2003 | 72.8 | 72.8 | 3115 (56.7) | 2379 (33.3) | 425 (7.73) | 427 (7.78) | 3482 (63.37) | 3027 (55.1) |
| Canivet (2014) [35] | 6530 | 7087 | 55 | 55 | 7742 (56.9) | 5875 (43.1) | - | - | - | - |
| Clark (2014) [36] | 155 | 1426 | 60 | 60 | 496 (31.2) | 1085 (68.3) | 90 (5.67) | 206 (13) | 62 (3.9) | 564 (35.5) |
| Hsu (2015) [37] | 22,040 | 22,040 | 47.7 | 47.7 | 25168 (57.1) | 18912 (42.9) | 3281 (14.9) | 3281 (14.9) | 5647 (25.6) | 5647 (25.6) |
| Gianfagna (2016) [38] | 267 | 2010 | 51.3 | 50.4 | 0 (0) | 2277 (100) | - | - | 62.2 (2.73) | 47.2 (2.07) |
| Choi (2017) [39] | 661 | 776 | 49.3 | 42.2 | - | - | 163 (3.86) | 265 (6.27) | 163 (3.9%) | 265 (6.27) |
| Lu (2018) [40] | 36,741 | 1,602,349 | 58.9 | 63.3 | 108267 (6.6) | 1530823 (93.4) | 6270 (0.38) | 348,062 (21.2) | 19,064 (1.16) | 928,677 (56.7) |
| Polanka (2019) [41] | 1838 | 1270 | 49 | 49 | 90 (2.9) | 3,018 (97.1) | - | - | - | - |
| Zheng (2019) [42] | 55127 | 432,073 | 53.2 | 50.8 | 287959 (59.1) | 199241 (40.9) | 29232 (6) | 25822 (5.3) | - | - |
| Lopes, 2021 [43] | 98 | 62 | 72.16 | 72.16 | 112 (71.8) | 44 (28.2) | 114 (73.1) | 42 (26.9) | 114 (73.1) | 42 (26.9) |
| sigurdardottir 2021 [44] | 844 | 7554 | 55.8 | 49.8 | 4614 (55) | 3784 (45) | 39 (0.46) | 201(2.4)) | 416(4.95) | 3191(38) |
| Lechat (2022) (1) [45] | 131 | 2214 | 61 | 61 | 2747 (52.5) | 2489 (47.5) | 8 (0.2) | 82 (1.97) | 58(1.4) | 617(14.8) |
| Mahmood (2022) [18] | 4252 | 9025 | - | - | - | - |  |  |  |  |
| Lechat (2022) (2) [20] | 170 | 2708 | 61 | 61 | - | - | 13 (0.2) | 138 (2.6) | 89 (1.7) | 990 (18.9) |
| Seko (2022) [46] | 330 | 3084 | 81 | 80 | 1528 (44.8) | 1886 (55.2) | 114 (3.3) | 1152 (33.7) | 237 (6.9) | 2243 (65.7) |
| Sambou (2022) [47] | 241,825 | 81,548 | 55 | 55 | 180,378 (55.8) | 142,931 (44.2) | - | - | - | - |
| Luu (2022) [21] | 7040 | 5408 | 43 | 43 | 1941 (16) | 10507 (84) | - | - | - | - |

**Supplementary Table 5:** Quality assessment of observational studies using Newcastle Ottawa Scale

| Study/Score | Selection | | | | Comparability | | Outcome | | |  | Rating |
| --- | --- | --- | --- | --- | --- | --- | --- | --- | --- | --- | --- |
|  | S1 | S2 | S3 | S4 | C |  | O1 | O2 | O3 | Total |  |
| Athius 1998 | * | * | * |  | * |  |  | * | * | 6 | Good |
| Schwartz 1998 | * | * |  | * | * | * | * | * |  | 7 | Good |
| Phillips 2007 | * |  | * | * | * |  | * | * | * | 7 | Good |
| Chien 2010 | * | * | * |  | * |  | * | * |  | 6 | Good |
| Jaussent 2013 | * | * |  | * | * |  | * | * |  | 6 | Good |
| Clarke 2014 | * | * | * | * | * | * | * | * |  | 8 | Good |
| Canvet 2014 | * | * | * | * | * |  | * | * |  | 7 | Good |
| Hsu 2015 | * | * | * |  | * |  | * | * | * | 7 | Good |
| Gianfagna 2016 | * | * | * | * |  | * | * | * | * | 8 | Good |
| Choi 2017 | * | * |  | * | * |  | * | * |  | 6 | Good |
| Lu 2018 | * | * | * |  | * |  | * | * | * | 7 | Good |
| Polanka 2019 | * | * | * | * | * | * | * | * | * | 9 | Good |
| Zheng 2019 | * | * | * | * | * |  | * | * | * | 8 | Good |
| Lopes 2021 | * | * | * | * | * |  | * | * |  | 7 | Good |
| Sigurdardottir 2021 | * | * | * |  | * |  | * | * |  | 6 | Good |
| Seko 2022 | * | * | * | * | * |  | * | * | * | 8 | Good |
| Lechat 2022 (1) | * | * | * |  | * | * | * | * |  | 7 | Good |
| Luu 2022 | * | * | * | * | * | * | * | * |  | 8 | Good |
| Lechat 2022 (2) | * | * |  | * | * | * | * | * | * | 8 | Good |
| Sambou 2022 | * | * | * | * | * | * | * | * |  | 8 | Good |
| Mahmood 2022 | * | * | * | * | * | * | * | * | * | 9 | Good |

**Supplementary Table 6:** Publication bias for outcomes

| Outcome | Bias Egger | Bias Begg |
| --- | --- | --- |
| MI | 0.4904 | 1.0000 |
| CV-mortality | 0.0662 | 0.2963 |
| All-cause mortality | 0.7585 | 0.4743 |
| CV-disease incidence | 0.2785 | 0.7545 |

**Supplementary Table 7:** Preferred Reporting Items for Systematic Reviews and Meta-analyses (PRISMA) checklist

| **Section and Topic** | **Item #** | **Checklist item** | **Location where item is reported** |
| --- | --- | --- | --- |
| **TITLE** | | |  |
| Title | 1 | Identify the report as a systematic review. | 1 |
| **ABSTRACT** | | |  |
| Abstract | 2 | See the PRISMA 2020 for Abstracts checklist. | 2 |
| **INTRODUCTION** | | |  |
| Rationale | 3 | Describe the rationale for the review in the context of existing knowledge. | 3-4 |
| Objectives | 4 | Provide an explicit statement of the objective(s) or question(s) the review addresses. | 3-4 |
| **METHODS** | | |  |
| Eligibility criteria | 5 | Specify the inclusion and exclusion criteria for the review and how studies were grouped for the syntheses. | 5 |
| Information sources | 6 | Specify all databases, registers, websites, organisations, reference lists and other sources searched or consulted to identify studies. Specify the date when each source was last searched or consulted. | 5 |
| Search strategy | 7 | Present the full search strategies for all databases, registers and websites, including any filters and limits used. | 5 |
| Selection process | 8 | Specify the methods used to decide whether a study met the inclusion criteria of the review, including how many reviewers screened each record and each report retrieved, whether they worked independently, and if applicable, details of automation tools used in the process. | 5 |
| Data collection process | 9 | Specify the methods used to collect data from reports, including how many reviewers collected data from each report, whether they worked independently, any processes for obtaining or confirming data from study investigators, and if applicable, details of automation tools used in the process. | 6 |
| Data items | 10a | List and define all outcomes for which data were sought. Specify whether all results that were compatible with each outcome domain in each study were sought (e.g., for all measures, time points, analyses), and if not, the methods used to decide which results to collect. | 6 |
|  | 10b | List and define all other variables for which data were sought (e.g., participant and intervention characteristics, funding sources). Describe any assumptions made about any missing or unclear information. | 6 |
| Study risk of bias assessment | 11 | Specify the methods used to assess risk of bias in the included studies, including details of the tool(s) used, how many reviewers assessed each study and whether they worked independently, and if applicable, details of automation tools used in the process. | 6 |
| Effect measures | 12 | Specify for each outcome the effect measure(s) (e.g., risk ratio, mean difference) used in the synthesis or presentation of results. | 7 |
| Synthesis methods | 13a | Describe the processes used to decide which studies were eligible for each synthesis (e.g., tabulating the study intervention characteristics and comparing against the planned groups for each synthesis (item #5)). | 7 |
|  | 13b | Describe any methods required to prepare the data for presentation or synthesis, such as handling of missing summary statistics, or data conversions. | 10 |
|  | 13c | Describe any methods used to tabulate or visually display results of individual studies and syntheses. | 7 |
|  | 13d | Describe any methods used to synthesize results and provide a rationale for the choice(s). If meta-analysis was performed, describe the model(s), method(s) to identify the presence and extent of statistical heterogeneity, and software package(s) used. | 7 |
|  | 13e | Describe any methods used to explore possible causes of heterogeneity among study results (e.g., subgroup analysis, meta-regression). | 7 |
|  | 13f | Describe any sensitivity analyses conducted to assess robustness of the synthesized results. | 7 |
| Reporting bias assessment | 14 | Describe any methods used to assess risk of bias due to missing results in a synthesis (arising from reporting biases). | 7 |
| Certainty assessment | 15 | Describe any methods used to assess certainty (or confidence) in the body of evidence for an outcome. | 7 |
| **RESULTS** | | |  |
| Study selection | 16a | Describe the results of the search and selection process, from the number of records identified in the search to the number of studies included in the review, ideally using a flow diagram. | 7 |
|  | 16b | Cite studies that might appear to meet the inclusion criteria, but which were excluded, and explain why they were excluded. | 9 |
| Study characteristics | 17 | Cite each included study and present its characteristics. | 7-8 |
| Risk of bias in studies | 18 | Present assessments of risk of bias for each included study. | 8 |
| Results of individual studies | 19 | For all outcomes, present, for each study: (a) summary statistics for each group (where appropriate) and (b) an effect estimates and its precision (e.g., confidence/credible interval), ideally using structured tables or plots. | 10-11 |
| Results of syntheses | 20a | For each synthesis, briefly summarise the characteristics and risk of bias among contributing studies. | 10-11 |
|  | 20b | Present results of all statistical syntheses conducted. If meta-analysis was done, present for each the summary estimate and its precision (e.g., confidence/credible interval) and measures of statistical heterogeneity. If comparing groups, describe the direction of the effect. | 10-11 |
|  | 20c | Present results of all investigations of possible causes of heterogeneity among study results. | 13-14 |
|  | 20d | Present results of all sensitivity analyses conducted to assess the robustness of the synthesized results. | 13-14 |
| Reporting biases | 21 | Present assessments of risk of bias due to missing results (arising from reporting biases) for each synthesis assessed. | 11-14 |
| Certainty of evidence | 22 | Present assessments of certainty (or confidence) in the body of evidence for each outcome assessed. | 12-13 |
| **DISCUSSION** | | |  |
| Discussion | 23a | Provide a general interpretation of the results in the context of other evidence. | 14-17 |
|  | 23b | Discuss any limitations of the evidence included in the review. | 17 |
|  | 23c | Discuss any limitations of the review processes used. | 17 |
|  | 23d | Discuss implications of the results for practice, policy, and future research. | 18 |
| **OTHER INFORMATION** | | |  |
| Registration and protocol | 24a | Provide registration information for the review, including register name and registration number, or state that the review was not registered. |  |
|  | 24b | Indicate where the review protocol can be accessed, or state that a protocol was not prepared. |  |
|  | 24c | Describe and explain any amendments to information provided at registration or in the protocol. |  |
| Support | 25 | Describe sources of financial or non-financial support for the review, and the role of the funders or sponsors in the review. |  |
| Competing interests | 26 | Declare any competing interests of review authors. |  |
| Availability of data, code and other materials | 27 | Report which of the following are publicly available and where they can be found: template data collection forms; data extracted from included studies; data used for all analyses; analytic code; any other materials used in the review. |  |

**Supplementary Table 8:** AMSTAR-2 (Assessing the methodological quality of systematic reviews-2) Guidelines checklist


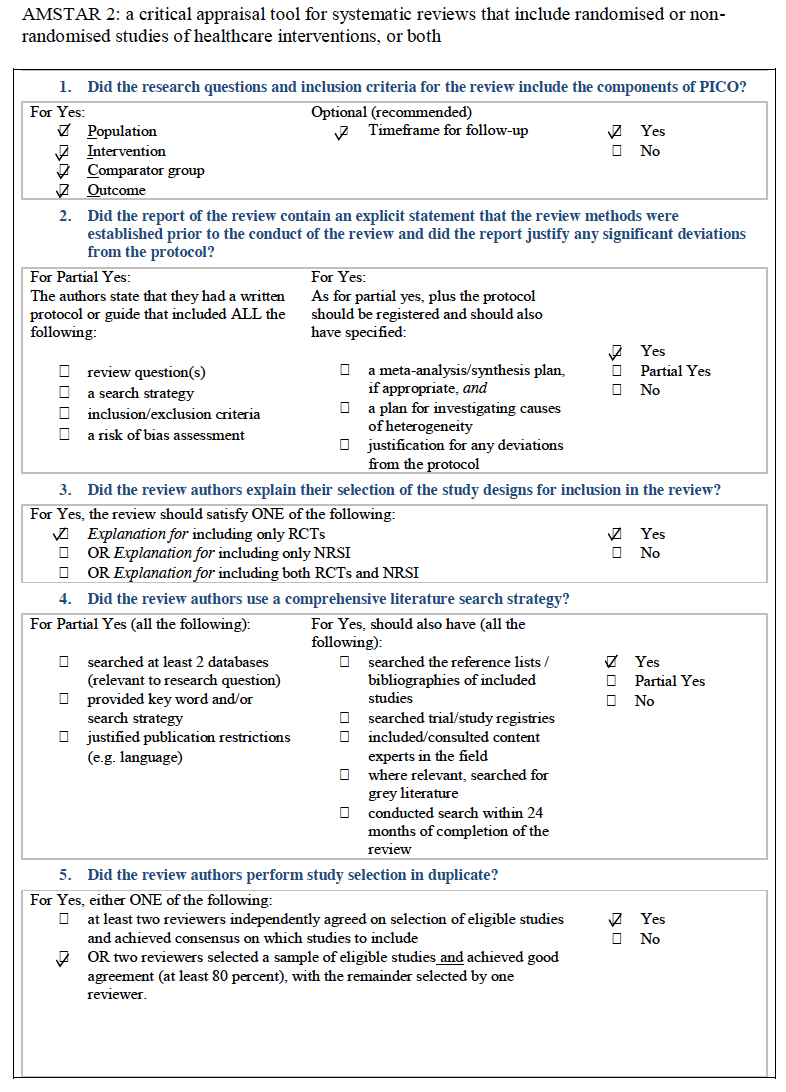


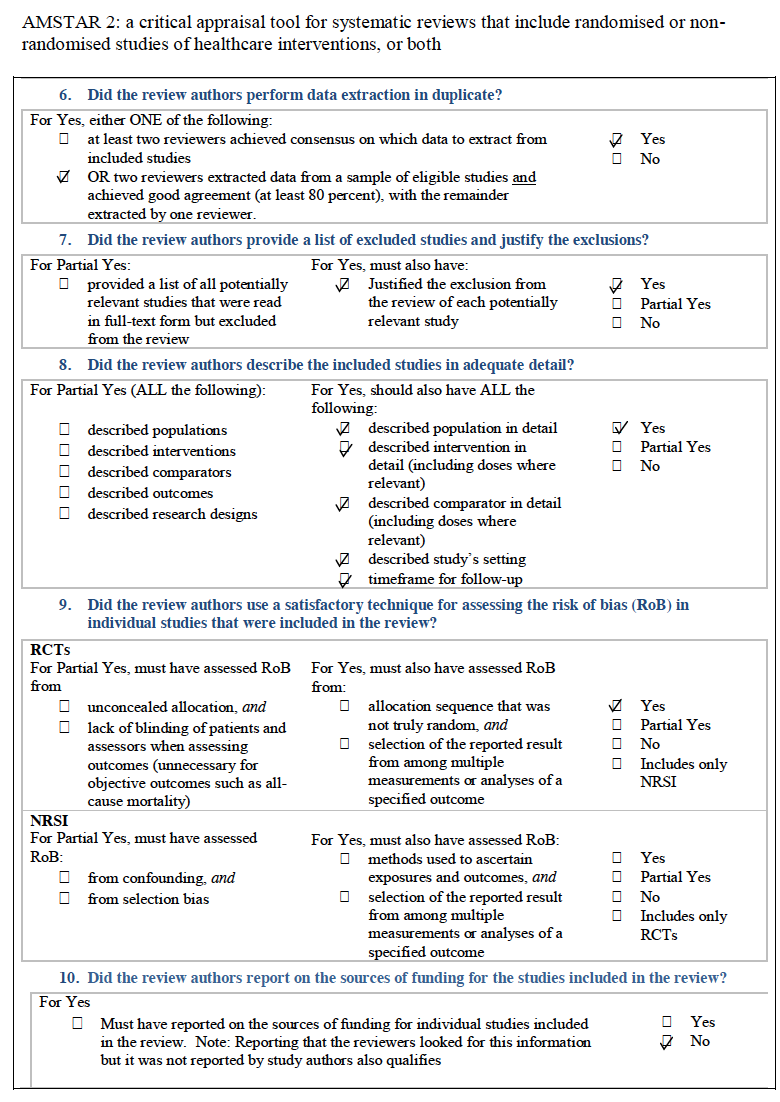


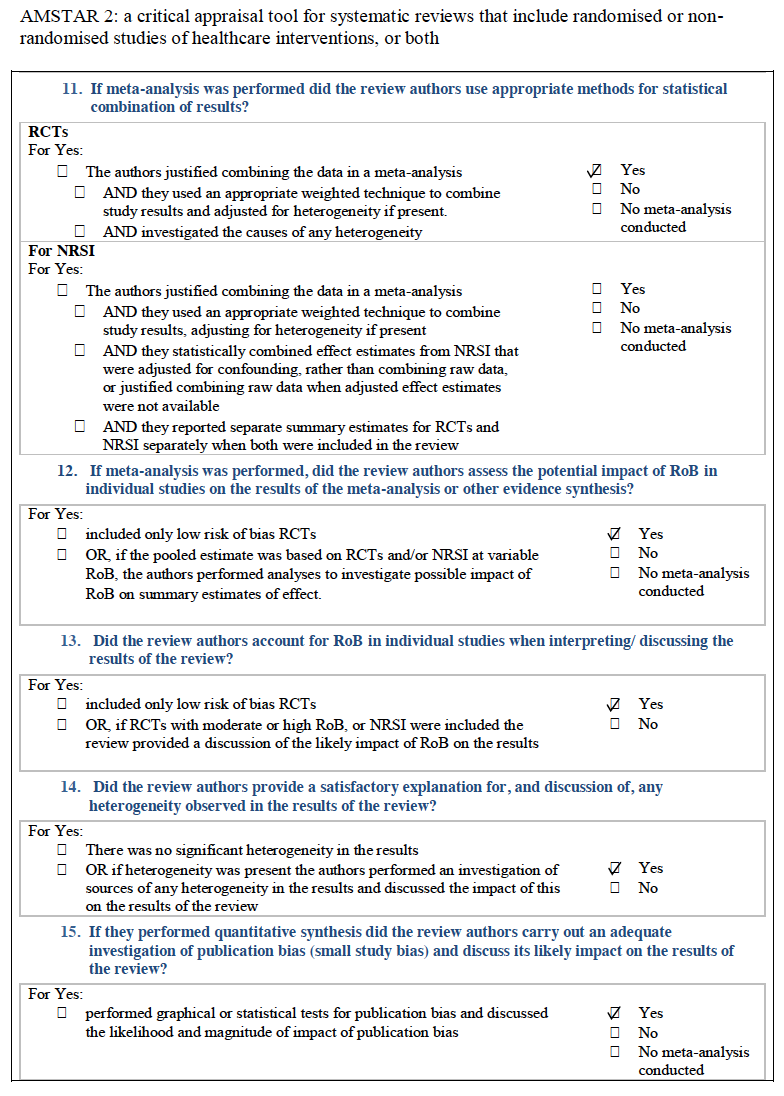


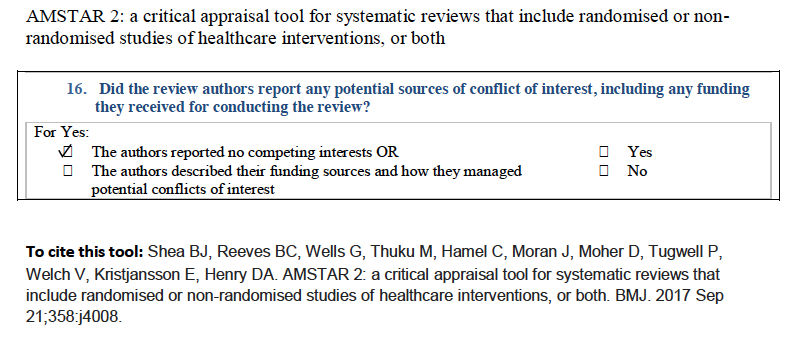


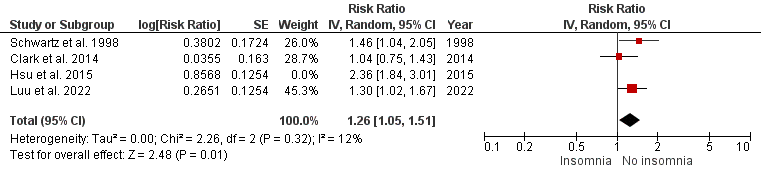


**Supplementary Figure 1:** Sensitivity analysis for MI after excluding Hsu et al. (2015)


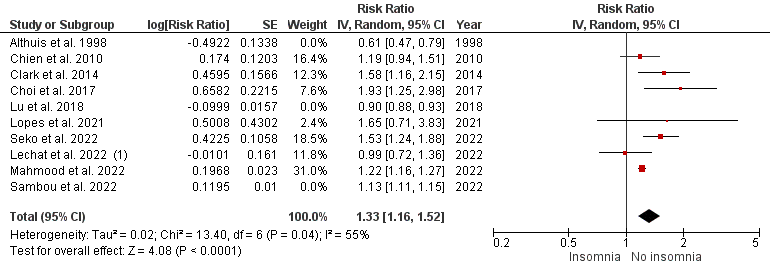


**Supplementary Figure 2:** Sensitivity analysis for all-cause mortality after excluding Sambou et al. (2022), Lu et al. (2018), and Althuis et al. (1998)


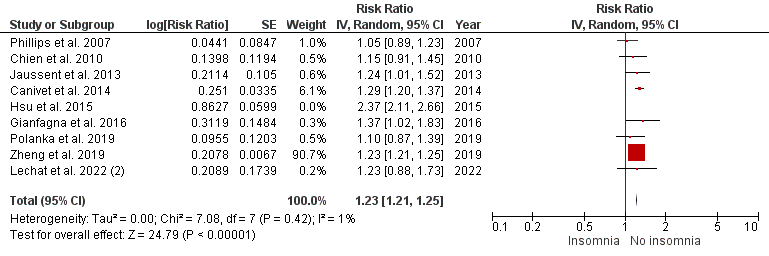


**Supplementary Figure 3:** Sensitivity analysis for CV-disease incidence after excluding Hsu et al. (2015)

**Funnel plots for primary and secondary outcomes**

**
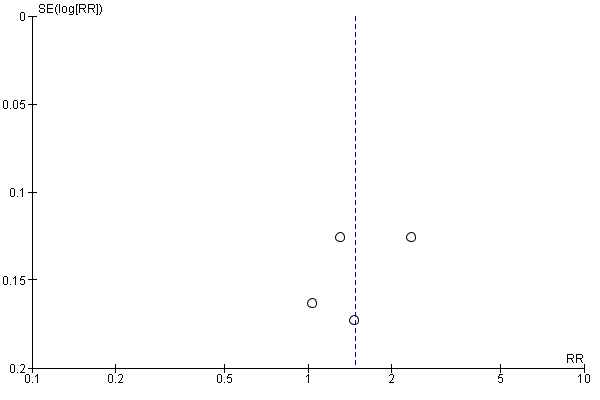
**

**Supplementary Figure 4:** Funnel plot for MI in insomnia vs without insomnia.

**
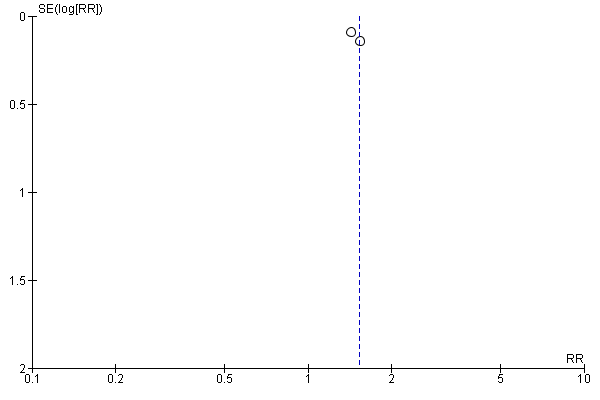
**

**Supplementary Figure 5:** Funnel plot for CV-mortality in insomnia vs. without insomnia.


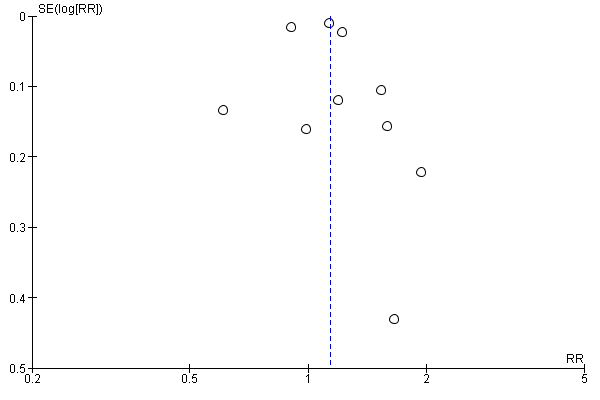


**Supplementary Figure 6**: Funnel pot for all-cause mortality in insomnia vs without insomnia.


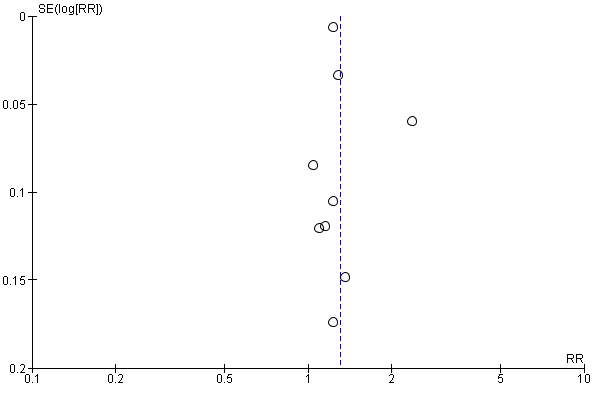


**Supplementary Figure 7**: Funnel plot for CV-disease incidence in insomnia vs. without insomnia.
